# Pelvic nerves during radical trachelectomy

**DOI:** 10.1136/ijgc-2020-001876

**Published:** 2020-12-28

**Authors:** Lei Li, Ming Wu

**Affiliations:** Obstetrics and Gynecology, Peking Union Medical College Hospital, Beijing, China

**Keywords:** uterine cervical neoplasms

**Figure 1:**
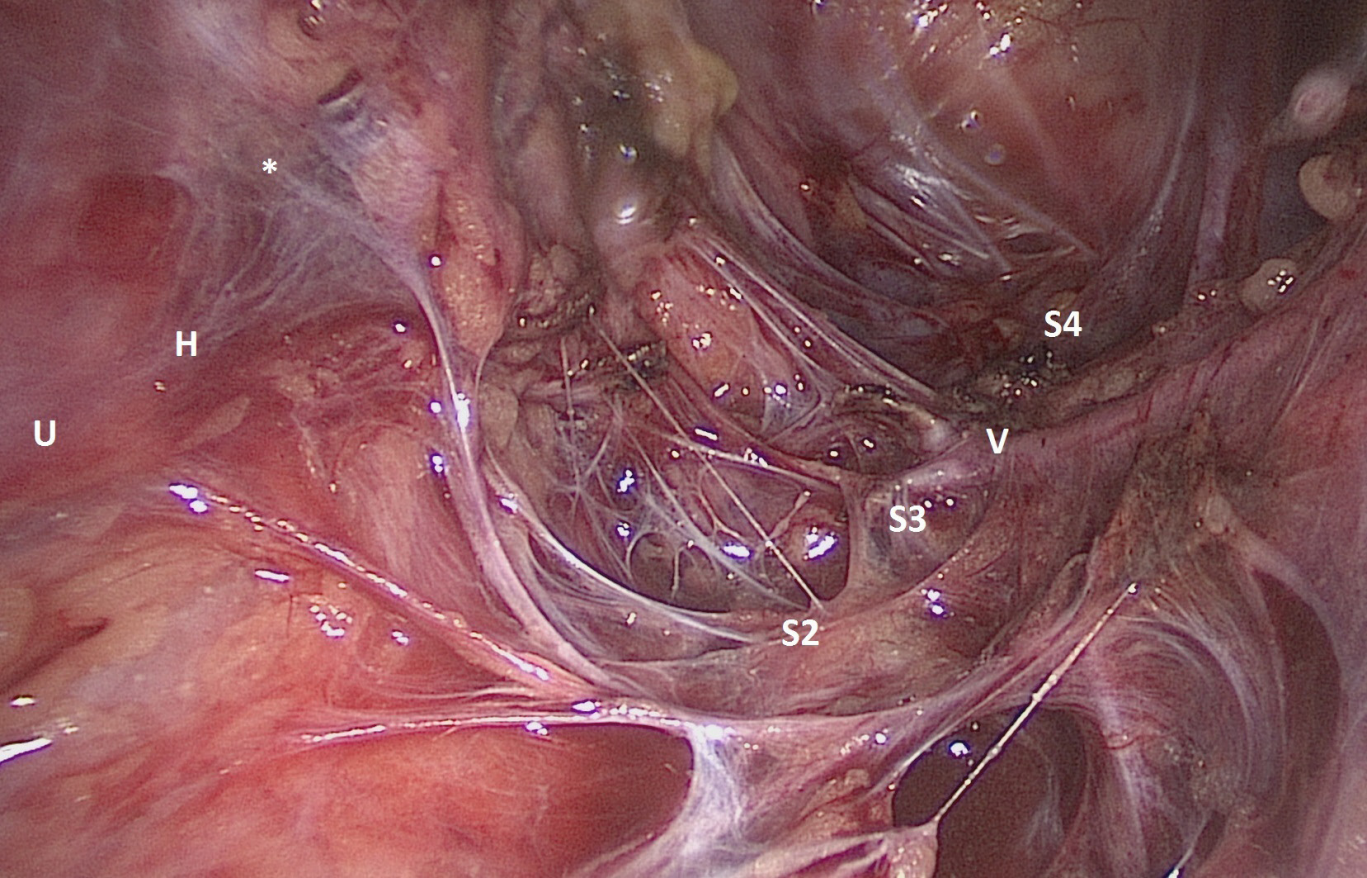
Right-side pelvic visceral nerves.*Inferior hypogastric plexus. H, hypogastric nerves; S2 to S4, second to fourth sacral nerves; U, right ureter; V, deep uterine vein.

A 24-year-old patient accepted laparoscopic radical trachelectomy for stage IB1 cervical squamous cell carcinoma. During the surgery(Figure 1), after resection of the deep uterine vein (V) and its branches by the right parametrium, the parasympathetic pelvic visceral nerves originating from sacral nerves (in this case, S2 to S4) are evident. long the right ureter (U), sympathetic hypogastric nerves (H) and pelvic visceral nerves are tangled up and constituted an inferior hypogastric plexus (that is, pelvic plexus). We demonstrate a type C1 surgery of Querleu-Morrow classification. Identification of the anatomy is critical for nerve-sparing surgeries.

